# Spatial and seroepidemiology of canine visceral leishmaniasis in an endemic Southeast Brazilian area

**DOI:** 10.1590/0037-8682-0525-2019

**Published:** 2020-05-18

**Authors:** Tamiris Fagundes Rodrigues, Aline do Nascimento Benitez, Anaiá da Paixão Sevá, Lucas Hidenori Okamura, André Batista Galvão, Jancarlo Ferreira Gomes, Katia Denise Saraiva Bresciani, Tereza Cristina Cardoso

**Affiliations:** 1 Universidade Estadual Paulista, Faculdade de Medicina Veterinária, Programa de Pós-Graduação em Ciência Animal, Araçatuba, SP, Brasil.; 2 Universidade de Campinas, Programa de Pós-Graduação em Saúde da Criança e do Adolescente, Campinas, SP, Brasil.; 3 Universidade de São Paulo, Departamento de Medicina Veterinária Preventiva e Ciência Animal, São Paulo, SP, Brasil.; 4 Universidade Estadual Paulista, Faculdade de Medicina Veterinária, Departamento de Produção e Saúde Animal, Araçatuba, SP, Brasil.

**Keywords:** Dual Path Platform, Kernel estimation, Leishmania, Risk factors, Zoonoses

## Abstract

**INTRODUCTION:**

Canine visceral leishmaniasis (CVL) is a public health problem, and its prevalence is associated with the coexistence of vectors and reservoirs. CVL is a protozoonosis caused by *Leishmania infantum* that is endemic in the southeast region of Brazil. Thus, vector and canine reservoir control strategies are needed to reduce its burden. This study aimed to verify the CVL seroprevalence and epidemiology in a municipality in Southeast Brazil to initiate disease control strategies.

**METHODS:**

A total of 833 dogs were subjected to Dual Path Platform (DPP) testing and enzyme-linked immunosorbent assays. For seropositive dogs, epidemiological aspects were investigated using a questionnaire and a global position system. The data were submitted to simple logistic regression, kernel estimation, and Bernoulli spatial scan statistical analysis.

**RESULTS:**

The overall CVL-confirmed seroprevalence was 16.08%. The 28.93% in the DPP screening test was associated with dogs maintained in backyards with trees, shade, animal and/or bird feces, and contact with other dogs and cats, with sick dogs showing the highest chances of infection (odds ratio, 2.6; 95% confidence interval, 2.38-1.98), especially in residences with elderly people. A spatial analysis identified two hotspot regions and detected two clusters in the study area.

**CONCLUSIONS:**

Our results demonstrated that residences with elderly people and the presence of trees, shade, feces, and pet dogs and cats increased an individual’s risk of developing CVL. The major regions where preventive strategies for leishmaniasis were to be initiated in the endemic area were identified in two clusters.

## INTRODUCTION

Visceral leishmaniasis (VL), or kala-azar, is a protozoonosis caused by *Leishmania infantum* inoculated by the bite of an infected phlebotomine sandfly^1^. Dogs (*Canis familiaris*), the only known reservoirs of this parasite, are responsible for the perpetuation of VL in such areas.

Environmental and cultural conditions are associated with infection prevalence in both reservoirs and hosts; however, the ability of the vector to infect different hosts and the close contact between owners and their pet dogs can influence the risk of VL infection^2^ ^-^ ^4^. In this scenario, the propagation of VL can be established by the high prevalence of dog seropositivity to canine VL (CVL).

Although many aspects of the ecoepidemiology of VL were discovered in the past 20 years, including its association with poor living conditions, as verified in developing countries^5^, the disease in humans and dogs can present high mortality rates if left untreated. The results of spatial analyses may improve public health actions for leishmaniasis since they can, for instance, be used to estimate the coverage of control measures for VL^6^ and predict the disease dispersion^7^.

Brazil is classified by the World Health Organization (WHO) as one of the six major countries of VL high-burden wolrdwide, which together account for approximately 90% of the global cases and expose 556 million people to the risk of infection^1^ ^,^ ^5^. In 2019, the WHO reported that VL is endemic in 12 countries in the Americas, with 59,769 new cases reported in 2001-2017, approximately 96% (57,582) of which were reported in Brazil^8^. The territorial dimension of the country is over 8,500,000 km², including two tropical biomes and the various fauna and flora of this climate in addition to the cultural diversity within the territories^9^.

Therefore, the governmental strategies developed to combat leishmaniasis address the epidemiological characteristics faced by each region and can vary among states. For example, in São Paulo, the disease has been present since 1999^10^, while in Paraná, to the best of our knowledge, there have been no reported cases in native reservoirs or humans^11^.

Worldwide, the leishmaniasis surveillance and control program recommended by the WHO is based on case detection and treatment combined with other health education measures, as well as taking action toward vectors and reservoirs when recommended^8^.

Despite these actions, in 2015, the Brazilian VL Control Program announced that epidemiological canine and human transmission conditions associated with the presence of *Lutzomyia longipalpis* could be verified in 82/645 (12.71%) São Paulo state municipalities, such as Piacatu, where the first human case of VL was recorded in 2008 in a 12-year-old child^5^ and two cases were recorded in 2010 in a 1-year-old child and a 4-year-old child. In 2017, 4,096 cases were reported through the Brazilian administrative states, with 147 (3.65%) in São Paulo state, of which 10/147 (6.80%) were fatal. In 2018, the mortality rate reached 8.76% (8/91)^12^. This framework suggests that current strategies to control the disease are insufficient^5^and efforts should be directed toward the major regions for public health interventions against VL.

Considering the importance of VL in public health, this study aimed to investigate the spatial epidemiological aspects and identify the spatial and descriptive aspects associated with the risk of CVL in the Piacatu/São Paulo municipality.

## METHODS

The Ethics Committee of FMVA School, UNESP (CEEA 2345/2014), approved the present study.

**FIGURE 1:**
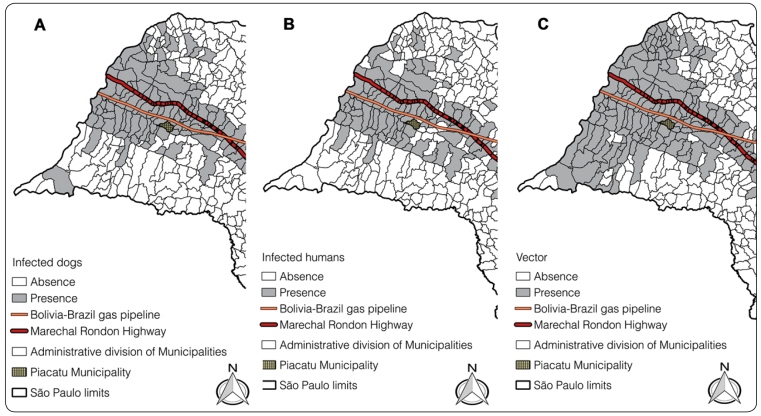
Piacatu location, São Paulo state, Brazil (2014). Representative pictures revealing a close view of the studied area location (dark grey) and Marechal Rondon highway and Brazil-Bolivia gas pipeline. Areas: **(A)** of infected dogs, **(B)** of infected humans, and **(C)** where *Lutzomyia longipalpis* vectors were found.

Piacatu is a municipality in the northwest region of São Paulo state (21° 35' 31" S, 50° 35' 56" W) with a population of 5,846 inhabitants and a total area of 232,488 km², of which 10% corresponds to urban areas and 90% to rural areas. The tropical climate is characterized by dry winters and rainy summers, with temperatures ranging from 18°C to ≥22°C^10^. The region is classified as endemic for VL, with reported cases in reservoirs and humans and the presence of the vector *Lutzomyia longipalpis* ^7^ (Figure 1). 

In 2014, the Piacatu Department of Zoonotic Disease Surveillance and Control, following instructions from the Department of Epidemiological Surveillance of the Ministry of Health, published the LV Surveillance and Control Manual and initiated disease characterization in urban areas using a fragmented strategy.

Four sequential phases were implemented: A) performing a canine census in all residences within the urban areas; B) inviting the animals’ tutors to participate in dog blood collection for anti-*L. infantum* serology; C) mapping of the blocks containing residences with seropositive dogs; and D) collecting epidemiological data using a questionnaire at all residences of that positive block.

**FIGURE 2:**
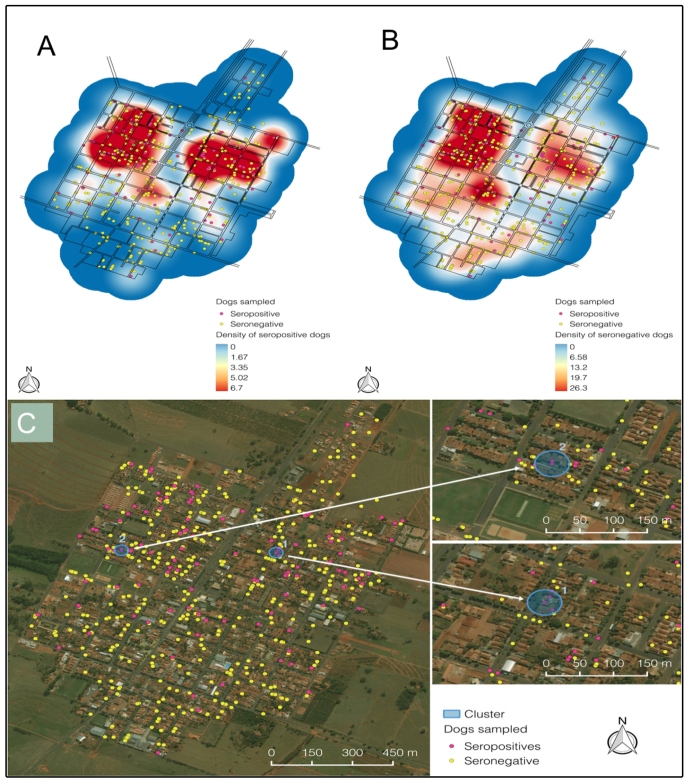
Spatial analysis of CVL in Piacatu, São Paulo, Brazil (2014). **(A)** Kernel estimate of dog seropositive population density by CVL. **(B)** Dog seronegative population density by CVL. Red indicates relatively high CVL risk, blue indicates relatively low risk. **(C)** Clusters (white circles with arrows head) of CVL risks inside Piacatu constructed based on high-high correlation of analyzed variables. CVL, canine visceral leishmaniasis.

In the canine census, all residences in the urban perimeter of the municipality (Figure 2) were individually visited to verify the presence of dogs within them. A house was included if any dog was recognized by the household as being in their care, either with restricted circulation in the indoor spaces and backyard or those with free access to the street. This study was limited since it could not obtain data on the population of stray dogs within the municipality.

All residences identified in phase A with pet dogs were included in this investigation for the serum collection in phase B, resulting in 833 serosamples. Sequential data were obtained from 647 dog tutors in phase D.

The dogs were restrained manually with their tutor’s support for blood collection performed after antisepsis with Alcohol 70° GL, followed by collection via cephalic venipuncture of up to 5 mL of material with a Vacutainer^®^ system in tubes with a separator gel and a clot accelerator.

After a 3-5-min rest, the flasks were sent to the Piacatu Municipal Zoonotic Control Center, where they were centrifuged for 10 minutes at 3,500 rpm for serum obtention. All serosamples were placed in sterile vials, identified, and stored at -20°C until use. The screening tests for CVL were performed with a Dual Path Platform (DPP^®^), and the seroreagents were tested at the Regional Brazilian Health Ministry Official Laboratory, Instituto Adolf Lutz, with enzyme-linked immunosorbent assay (ELISA) according to the manufacturer’s instructions (Biomanguinhos^®^).

Data were obtained from all dog tutors after they signed an informed consent form. Questions included those pertaining to the characteristics of the backyard based on vector (*Lu. longipalpis*) behavior and the presence of pet animals. The presence of animal feces in the backyard, trees, or shadows and the use of a repellent collar by the dog were obtained from the interviewer’s visualization. The knowledge was considered positive if at least one characteristic of prevention, transmission, and symptoms was correctly reported by the household. Other questions reported by the household included age; presence of fever, weakness, and/or discomfort; presence of children 0-12 years old in the residence; canine weight loss, onicography, alopecia, and/or wounds; and presence of stray and/or pet cats and/or dogs and/or fowl in the backyard. 

The data were analyzed with simple logistic regression in which the odds ratio was obtained by exponentiation of the regression coefficient. Associations were considered significant if the probability value was lower than α = 0.05/N. Considering a 95% confidence interval and a margin of error of 16%, the estimated prevalence of CVL was 13.6-18.6%.

All maps and kernel estimations were performed with the QGIS^®^ program, version 2.8, to identify hotspot regions in the studied area^13^ ^,^ ^14^. We used the Satscan™ program, version 9.4.2, to perform a Bernoulli spatial scan to identify clusters of positive animals^14^.

## RESULTS

Anti-*Leishmania* spp. antibodies were observed in 28.93% (241/833) of the animals using the DPP test and confirmed in 55.6% (134/241) using ELISA. Overall, 16.08% (134/833) of the dogs were seropositive for CVL in the municipality.

Kernel analysis revealed a small difference in the dispersion of cases in the urban area of Piacatu, in which the seropositive cases showed a slight concentration in relation to the seronegative cases (Figures 1A, 1B). Two clusters were detected and considered high-high risk areas (relative risk, >6.0; p < 0.05) for CVL. Moreover, a spatial correlation was observed for seropositive dogs and environmental conditions, such as backyards with trees, birds, feces, and shade (Table 1).

**TABLE 1:** Simple logistic regression and odds ratio of variables associated with the risk of canine visceral leishmaniosis infection in 647 households in Piacatu/São Paulo, from January to March 2014.

Variable	OR (95% CI)	P
Feces*	2.021 (1.18-3.46)	0.0104
Trees*	2.612 (1.66-4.08)	2.64425E^-05^
Shadow*	2.389 (1.50-3.80)	0.0002
Chickens*	1.987 (1.16-3.40)	0.0123
Elderly people*	1.575 (1.02-2.41)	0.037
Dogs*	3.155 (1.98-5.01)	1.15301E^-06^
Cats*	2.830 (1.76-4.52)	1.44017E^-05^
Dog with clinical signs*	1.843 (1.18-2.86)	0.007
Repellent collars	1.089 (0.44-2.66)	0.852
Children	0.816 (0.37-1.77)	0.607
Visceral leishmaniosis definition knowledge	0.891 (0.40-1.94)	0.771
Human symptoms	0.676 (0.26-1.75)	0.422
Visceral leishmaniosis prevention knowledge	1.209 (0.78-1.86)	0.390

*Probability value lower than α = 0.05/N.

## DISCUSSION

The 16.08% CVL infection verified in the Piacatu municipality according to the Brazilian Ministry of Health confirmed the transmission of *L. infantum* among reservoirs and suggested the possibility of human infection in the area.

Depending on the geographical conditions, climate, and social aspects of each affected region, seroprevalences of 4-75% have been reported in Brazilian territories; therefore, the prevalence verified in this study is within the range of those expected for endemic areas^7^.

The risk of canine infection was associated with residences with backyards with trees, chickens, shade, animal feces, dogs and/or cats, and dogs with clinical signs of CVL, especially when elderly people were present at the residence.

In Brazil, one can easily find regions with both rural and urban environments that show faunal and floral diversity among geographical regions; however, the presence of abundant vegetation along sidewalks and in gardens and backyards provides conditions favorable for vector maintenance^15^. This mixed urban-rural characteristic may explain the association verified by this study.

Here, we observed that feces had a 2.02× greater chance of contributing to CVL prevalence than the other measured backyard characteristics. Relationships with backyard characteristics were cited as having an associated risk of VL in another Brazilian study, and studies verified that dogs sleeping in yards were more likely to be infected than those with free access to the house^16^.

There is a contradiction in the association between green surroundings, trees, and shade and the occurrence of CVL^7^ ^,^ ^17^ ^,^ ^18^. These areas are rich in the organic substrates that are required for vector reproduction, as *Lu. longipalpis* has limited dispersal capacity because they cannot fly beyond 243 m^19^. Thus, a close proximity to vegetated areas may be associated with the risk of CVL^4^. However, in endemic regions, this association is compromised because reservoir presence is common in the in-home environment and urban afforestation offers conditions for phlebotomine sandfly reproduction^20^ ^,^ ^21^.

The association of chickens and/or other birds with CVL-positive cases has resulted in controversial studies with no statistical significance^22^ ^,^ ^23^. An epidemiological review of CVL in Brazil suggested a possible route of parasite transmission between chickens and humans^22^. It has been verified that the continuous risk of transmission of *L. infantum* depends heavily on the chicken blood present in the peridomiciliary environment since *Leishmania* vectors show a predilection for this animal. Its DNA was detected in 98.3% of the vectors captured in the peridomiciliary area, whereas parasite DNA was detected in 64.9%^24^. A similar result was obtained in Dracena, located 131 km from Piacatu, where concentrations of up to 5 × 10³ parasites in each vector were captured in a kennel, rural residence, urban area, and chicken coop^25^.

This fact, together with the geographical location of Piacatu along the border of the Brazil-Bolivia gas pipeline and near the Marachal Rondon Highway, corroborates the results of another study that demonstrated the dispersion of VL in the state of São Paulo (Figures 2A-2C). Through the results of the same study conducted in every state, it is possible to verify that the city of Piacatu is inserted between the municipalities presenting the vector *Lu. longipalpis* (Figure 2A) and cases of leishmaniasis in dogs (Figure 2B) and in humans (Figure 2C)^4^ ^,^ ^16^ ^,^ ^23^ ^,^ ^26^.

Although it has been reported that human cases tend to be located where sick dogs are located, a review of the literature of the importance of animals with CVL to their owners reported no consensus regarding the association between the culling of infected dogs^7^ or the seroprevalence of CVL^6^ and the prevalence of the disease in humans, suggesting that further studies should evaluate the impact of canine disease.

The presence of pets, such as dogs and/or cats, in residences provides conditions for *L. infantum* to complete its life cycle in the canine reservoir, with cats serving as accidental hosts. The presence of these pets in residences and the association with the risk of VL development agree with other reports of a higher CVL rate in dogs in contact with other dogs^8^ ^,^ ^9^ and in cats cohabiting with dogs previously affected by VL^27^.

No report to date has examined the relationship between CVL prevalence and elderly individuals^7^ ^,^ ^23^ ^,^ ^26^. In fact, this study reported for the first time that elderly people living at home have a 1.57-fold greater chance of contributing to CVL occurrence than that observed among homes without elderly individuals. However, the global role of elderly people in CVL epidemiology requires further analysis. This result suggests that health education should be intensified in regions with a high density of residences with elderly persons. The health education activities for that population require methods attempting to address the complexity of the aging process and its associated factors, such as values, beliefs, norms, and ways of life^28^.

Additionally, the spatial kernel analysis demonstrated concentrations of CVL cases in two areas in the urban area, where two clusters were detected. The mapping of positive CVL cases in this study provided a broader analysis of the spatial distribution and areas with a high prevalence of infected animals. Likewise, other researchers have successfully used this tool to develop public health surveillance strategies^19^ ^-^ ^21^ ^,^ ^24^ ^,^ ^29^.

The analysis performed in this study indicated that several factors are associated with the disease prevalence in a given region; similarly, a study conducted in India showed the spatial distribution of VL cases, making it possible to verify the association between hotspots and poverty^30^. Another study conducted in a rural area of Madrid where there was an outbreak of VL verified the spatial association between cases and the presence of vectors and rodents in the family Leporidae^31^.

The association of clusters with environmental variables suggests that, in this city, the disease behavior may be associated with the ecology of the phlebotomine sandfly; thus, it is possible that the focus of public health measures in environmental education can reduce the number of cases in dogs and, therefore, humans. These results corroborate those obtained in the city of Araçatuba, where the researchers reported transmission patterns of *Leishmania* of up to 45.7 m between cases, most likely related to *Lu. longipalpis* characteristics^6^.

After the high prevalence and spatial dispersion of CVL were verified in this study, an epidemiological task force was established focusing on health education for elderly people promoting backyard and street cleaning to remove organic waste produced by afforestation and chicken rearing.
